# Temporal Parameters Estimation for Wheelchair Propulsion Using Wearable Sensors

**DOI:** 10.1155/2014/645284

**Published:** 2014-07-03

**Authors:** Manoela Ojeda, Dan Ding

**Affiliations:** VA Pittsburgh Healthcare System, 6425 Penn Avenue, Suite 400, Pittsburgh, PA 15206, USA

## Abstract

Due to lower limb paralysis, individuals with spinal cord injury (SCI) rely on their upper limbs for mobility. The prevalence of upper extremity pain and injury is high among this population. We evaluated the performance of three triaxis accelerometers placed on the upper arm, wrist, and under the wheelchair, to estimate temporal parameters of wheelchair propulsion. Twenty-six participants with SCI were asked to push their wheelchair equipped with a SMART^Wheel^. The estimated stroke number was compared with the criterion from video observations and the estimated push frequency was compared with the criterion from the SMART^Wheel^. Mean absolute errors (MAE) and mean absolute percentage of error (MAPE) were calculated. Intraclass correlation coefficients and Bland-Altman plots were used to assess the agreement. Results showed reasonable accuracies especially using the accelerometer placed on the upper arm where the MAPE was 8.0% for stroke number and 12.9% for push frequency. The ICC was 0.994 for stroke number and 0.916 for push frequency. The wrist and seat accelerometer showed lower accuracy with a MAPE for the stroke number of 10.8% and 13.4% and ICC of 0.990 and 0.984, respectively. Results suggested that accelerometers could be an option for monitoring temporal parameters of wheelchair propulsion.

## 1. Introduction

According to the 2010 Survey of Income and Program Participation (SIPP), about 3.6 million people aged 15 years and older in the USA use a wheelchair [1]. Most of these individuals use a manual wheelchair for mobility [2]. Manual wheelchair users often rely on their upper extremities for almost all activities of daily living (ADLs). Some of their daily activities such as wheelchair propulsion and transfers require high forces and repetitiveness of upper extremities movements. Therefore, it is not surprising that the incidence of upper extremity pain and injury among manual wheelchair users is high, ranging from 49% to 78% [3–11].

Given the negative impact that upper extremity pain and injury may have on the lifestyle and quality of life of manual wheelchair users [9, 12–14], the Consortium for Spinal Cord Medicine published the monograph,* Preservation of Upper Extremity Function Following Spinal Cord Injury: A Clinical Practice Guideline for Health Care Professionals*, where it provides concise ergonomic and equipment recommendations based on the review of published evidence [15]. The guideline recommends reducing the frequency of repetitive upper limb tasks, minimizing forces required to complete tasks, and minimizing extremes of wrist and shoulder motions. It also makes recommendations on wheelchair propulsion techniques such as reducing the stroke number and push frequency. 

Temporal parameters of wheelchair propulsion such as the stroke number and push frequency have been quantified in laboratory settings using motion capture systems and SMART^Wheels^ (Three Rivers Holdings, LLC) a force sensing wheel that can replace the wheelchair wheel to collect propulsion parameters [5, 16–18]. Unfortunately, due to the cost and intricate settings, these valuable tools are not appropriate for assessing upper extremity movement in the home and community environment. Therefore, the repetitiveness of upper extremity movement for wheelchair propulsion out of clinical settings is unclear. With the recent advancement of sensors and miniature technologies, accelerometers emerge as a possible solution for monitoring wheelchair propulsion parameters, potentially contributing to the understanding and prevention of upper extremity pain and injury among manual wheelchair users. 

Previous studies have used accelerometers and other sensors to track gross mobility of manual wheelchair users. A pilot study conducted by Kumar et al. used a customized data-logging device to determine driving characteristics including distance, speed, and driving time of 19 power wheelchair soccer players [19]. A similar study conducted by Coulter et al. used two triaxial accelerometers placed on the wheels of a wheelchair to estimate gross mobility of 14 manual wheelchair users with spinal cord injury (SCI). The results showed that the accelerometers were able to recognize wheelchair propulsion episodes with an overall accuracy of 92% [20]. A study conducted by Gendle et al. investigated the revolutions, duration, and direction of movements. They found that the activity counts from the accelerometer were significantly different between light and moderate effort [21]. Other researchers have evaluated the performance of accelerometers in detecting manual wheelchair users' activities. A study conducted by Postma et al. used six two-axis accelerometers placed around the wrists, thighs, and along the sternum, respectively, to detect wheelchair propulsion episodes and its intensity from a range of ADLs among 10 manual wheelchair users. All six accelerometers were wired to a data recorder attached to the waist. Wheelchair propulsion episodes were identified using a wheelchair detection knowledge based on different body postures. The study showed that the six accelerometers were able to detect wheelchair propulsion episodes with an overall agreement of 92%. However, having 6 accelerometers on the body may prevent the user from moving freely in a natural environment [22]. Despite the fact that gross mobility and its intensity are, to some extent, indicative of manual wheelchair users' upper extremity movements, they cannot tell the exact amount and repetitiveness of upper extremity movements for wheelchair propulsion. 

Knowing the repetitiveness of upper extremity movements for wheelchair propulsion that occur on a daily basis could be important for understanding and preventing upper extremity pain and injury. However, research looking into using wearable sensors to directly estimate temporal parameters of wheelchair propulsion is limited. A study conducted by Koontz et al. estimated temporal parameters of wheelchair propulsion including push time, propulsion time, and recovery time based on hand acceleration collected via a 6-camera VICON motion analysis system among 29 manual wheelchair users. Position of the third metacarpal phalangeal joint was converted into instant velocity and instant acceleration. Push, propulsion, and recovery time were estimated by detecting acceleration sign change. Estimated parameters were compared with temporal parameters obtained from the SMART^Wheels^ (Three Rivers Holdings, LLC). Results showed high intraclass correlation between the estimated and criterion measures [23]. This study showed the feasibility of using hand acceleration to determine propulsion parameters. However, the hand acceleration in this study was derived from the 6-camera VICON system instead of a wearable accelerometer. A study conducted by Turner investigated the use of an accelerometer placed beneath the chair and a wheel-mounted magnet to detect wheelchair propulsion parameters including the stroke number, push frequency, distance, and speed. Ten manual wheelchair users were asked to propel their wheelchair on indoor and outdoor surfaces. Estimated parameters were compared with criterion values obtained from OptiPush wheels. Results showed the average percentage of errors were −1.0% for the stroke number and −1.7% for push frequency [24].

The purpose of this study is to assess the validity of a triaxis accelerometer placed at three locations (i.e., wrist, upper arm, and underneath the wheelchair seat) in estimating temporal parameters of wheelchair propulsion including the stroke number and push frequency. The information obtained can guide the use of accelerometers for monitoring temporal parameters and upper extremity movements during wheelchair propulsion.

## 2. Material and Methods

### 2.1. Study Participants

The Institutional Review Board at the University of Pittsburgh approved this study. A total of 26 manual wheelchair users with SCI volunteered and provided informed consent prior to their participation in the study. Subjects were identified through the IRB approved wheelchair user registries developed by the Human Engineering Research Laboratories (HERL) and the Department of Physical Medicine and Rehabilitation at the University of Pittsburgh. Subjects were included in the study if they (1) were 18 years of age or greater; (2) use a manual wheelchair as a primary means of mobility; and (3) have SCI. Subjects were excluded if they were unable to tolerate sitting for 2 hours and/or have upper limb pain that limits their mobility.

### 2.2. Instrumentation

**Figure 1:**
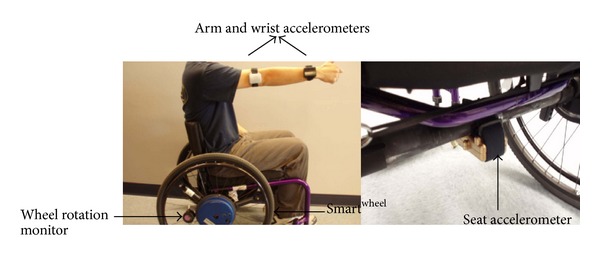
Instrumentation setup.

Subjects were fitted with four monitoring devices and a SMART^Wheels^ (Three Rivers Holdings, LLC). As shown in Figure 1, the four monitoring devices included a custom wheel rotation monitor attached to the wheelchair wheel and three off-the-shelf triaxis accelerometers (Shimmer Research, Dublin) worn on the dominant upper arm, dominant wrist, and underneath the wheelchair seat, respectively.

- The wheel rotation monitor was developed at the HERL. It is a lightweight and self-contained device that can be easily attached to the wheelchair's wheel without any modifications to the wheelchair. It tracks the wheel rotation through three reed switches mounted 120° apart on the back of the printed circuit board and a magnet mounted at the bottom of a pendulum. As the wheel rotates and exceeds 120° of rotation, one of the reed switches is triggered, and a date and time stamp is recorded. This information can be further processed to obtain the distance, speed, and time of movement [25]. The wheel rotation monitor has been used in previous studies to collect mobility characteristics of manual wheelchair users with different diagnoses [19, 26, 27].
- The triaxis accelerometer (Shimmer Research, Dublin) used in this study is a small low-power device that can record the motion data into a micro SD card. The two upper arm accelerometers were sampled at 20 Hz and the accelerometer underneath the seat was sampled at 60 Hz.
- The SMART^Wheels^ (Three Rivers Holdings, LLC) is a 3D force and torque-sensing wheel that measures push forces, push smoothness, push frequency, speed, and push length in every push cycle. It is sampled at 240 Hz. Subjects' wheelchair wheels were replaced with a SMART^Wheels^ (Three Rivers Holdings, LLC) at the dominant side and a dummy wheel at the other side to balance the weight of the SMART^Wheels^ (Three Rivers Holdings, LLC). The use of SMART^Wheels^ (Three Rivers Holdings, LLC) did not change the camber or the axle position.

### 2.3. Experimental Protocol

Subjects were asked to pay two visits to HERL with each visit lasting about 2.5 hours. During the first visit, subjects completed a demographics survey and the Wheelchair Users Shoulder Pain Index Questionnaire (WUSPI). The WUSPI questionnaire measures shoulder pain based on 15 questions using a 10 cm visual analogue scale, resulting in a total score from 0 (no pain) to 150 (extreme pain) [28]. After subjects were fitted with the instrumentation described in the previous section, they were asked to propel their own wheelchairs on two surfaces including a level surface of 33 meters and a sloped surface of 15 meters with 5 degrees of incline. A total of 24 level-surface trials performed at self-selected speed, low speed, and fast speed, and 12 sloped-surface trials at a self-selected speed were completed by each subject. All trials were videotaped using a hand-held digital video recorder.

During the second visit, participants were first asked to perform the propulsion trials as detailed for the first visit. Participants were then asked to complete a training session where they watched a multimedia instructional program on a laptop computer that aimed to teach appropriate propulsion techniques. The multimedia instructional program was developed by a previous study based on propulsion biomechanics literature and the Clinical Practice Guideline, which emphasized reducing push frequency and increasing push angle [29]. Examples of good and bad techniques were provided. After subjects practiced the propulsion techniques following the video training, they were asked to perform the same propulsion trials. This visit allowed us to assess if the accelerometers were capable of capturing propulsion changes due to training.

### 2.4. Data Collection and Analysis

Videos recorded during the two visits served as the criterion measure of the stroke number. Two investigators independently counted the stroke number for each propulsion trial, and video footages were reexamined when there was a discrepancy between the two investigators. The criterion push frequency was directly obtained from the SMART^Wheels^ (Three Rivers Holdings, LLC).

**Figure 2:**
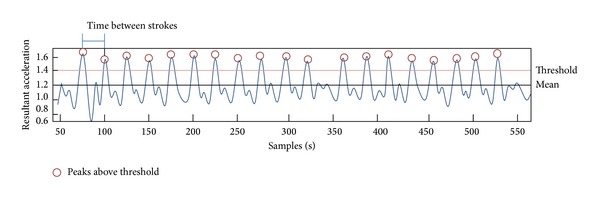
Visual example for stroke number and push frequency estimation.

Data from the wheel rotation monitor was converted to the wheel speed, which was used to identify wheelchair propulsion episodes and segment the acceleration data for each trial. Acceleration signals obtained from the accelerometers on the wrist, upper arm, and underneath the seat were filtered to remove high frequency noise using an 8th-order Butterworth low-pass filter. Butterworth filters have response characteristics that are appropriate for filtering wheelchair propulsion kinematic data as shown in previous studies [30–32]. Butterworth filters are commonly used to filter noisy signals because they introduce almost no distortion on the pass band while zeroing the noise on higher frequencies. A higher order filter (8th-order) was used to narrow the transition bandwidth which is wide in this type of filters [33]. The cutoff frequency was defined by the fundamental frequency calculated based on each propulsion trial with values ranging from 2 to 6 Hz. For the arm and wrist accelerometers, the resultant accelerations (the vector sum of three directions) were used to obtain the stroke number. For the seat accelerometer, only the longitudinal component (parallel to the propulsion direction) was used. An algorithm was developed to extract the stroke number from each propulsion trial. The algorithm first calculated a threshold defined as the mean acceleration plus 0.5 standard deviation over each trial. The stroke number was then counted as the number of acceleration peaks over the established threshold. Push frequency was calculated as the mean propulsion time between each two consecutive strokes.  Figure 2 shows a visual example of the stroke number and push frequency estimation.

Custom MATLAB (Version 7.11.0 R2010b, The Mathworks, Inc., USA) programs were used to process the acceleration signals.

The estimated stroke number and push frequency from the three accelerometers were compared with the criterion by calculating the mean absolute error (MAE) which was calculated as the average of the absolute difference between the estimated and the criterion, and mean absolute percentage of error (MAPE) calculated as the average ratio between the absolute difference and the criterion MAE = (1/*n*)∑_*i*=1_^*n*^|*E*_*i*_ − *C*_*i*_| and MAPE = (1/*n*)∑_*i*=1_^*n*^(|*E*_*i*_ − *C*_*i*_|/*C*_*i*_), where *E*_*i*_ is the estimated measure and *C*_*i*_ is the criterion measure. In addition, the intraclass correlation coefficients (ICC 3, 1) were used to assess their agreements. Bland-Altman plots were performed to provide a visual analysis of their agreements. Each point on the Bland and Altman plot represents the mean (*x*-axis) and the difference (*y*-axis) of the criterion and estimated values for each propulsion trial [34]. Propulsion trials during the first and the second visit were compared to assess the agreement between the estimation and the criterion.

Intraclass Correlation Coefficients (ICC 3, 1) were calculated to assess the validity of the accelerometers in detecting changes after training. Independent paired *t*-test was performed to evaluate significant differences before and after training. All statistical analysis was performed using SPSS software (ver. 18.0, SPSS Inc., Chicago, IL, USA).

## 3. Results

**Figure 3:**
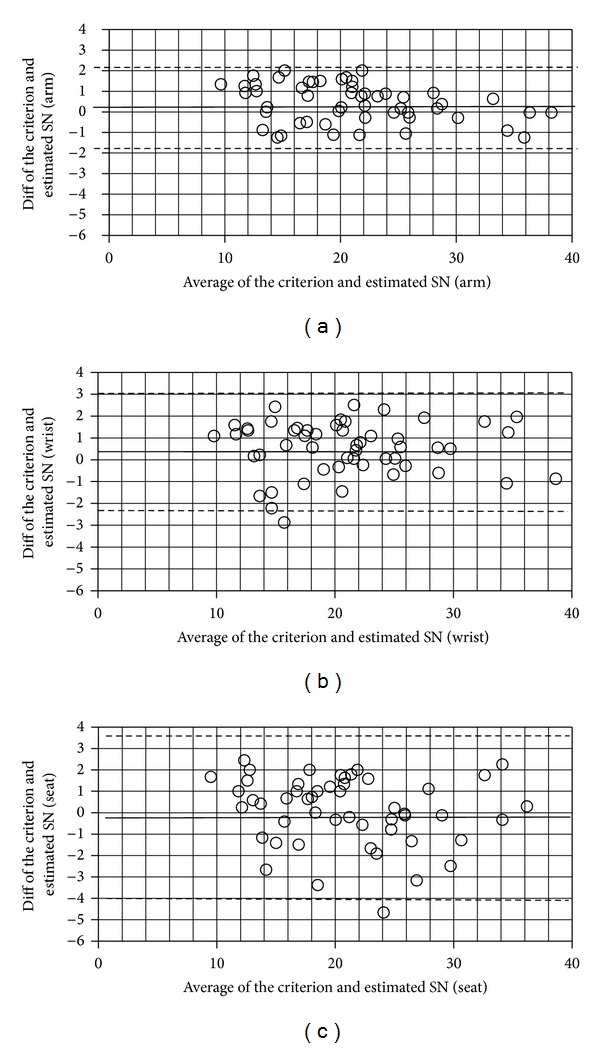
Stroke number Bland-Altman plots from the arm (a), wrist (b), and seat (c) accelerometers.

**Figure 4:**
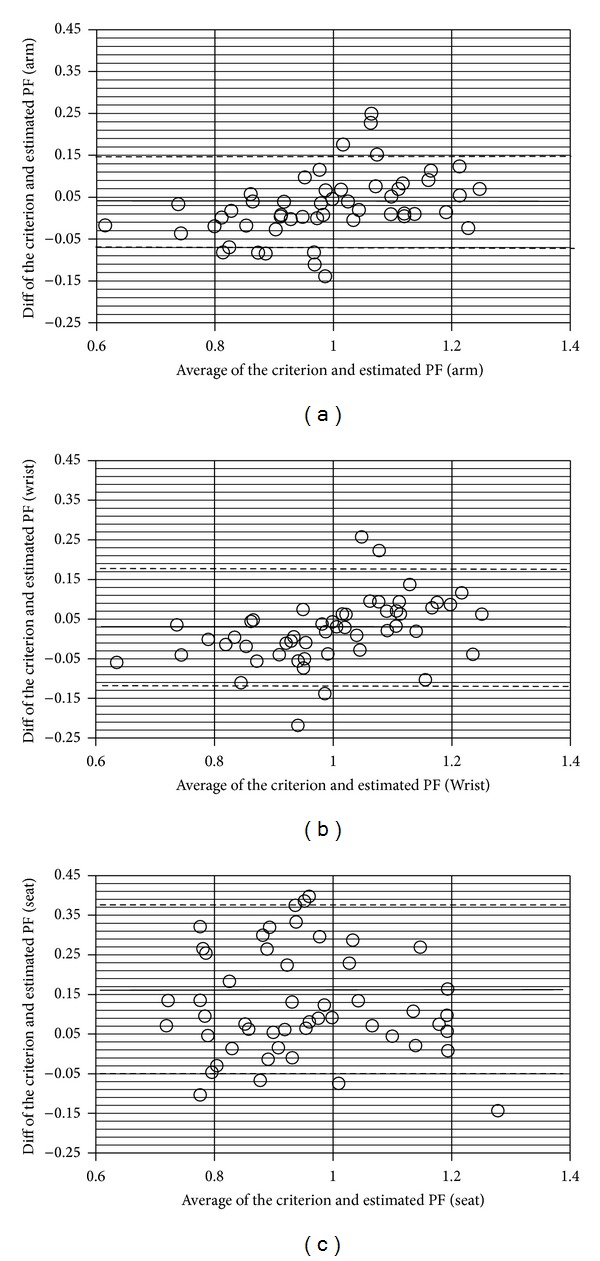
Push frequency Bland-Altman plots from the arm (a), wrist (b), and seat (c) accelerometers.

The demographics of the participants are described in Table 1. Tables 2 and 3 show the mean and standard deviation of the criterion and estimated stroke number and push frequency.  Table 4 shows the MAE and the MAPE between the criterion and estimated stroke number from each accelerometer.  Table 5 shows the MAE and MAPE between the criterion and estimated push frequency from each accelerometer.  Table 6 shows the ICC (3, 1) between the criterion and estimated temporal parameters for each accelerometer.  Table 7 shows the criterion and estimated stroke number before and after training, changes, and *P* values.  Table 8 shows the criterion and estimated push frequency before and after training, changes, and *P* values.  Table 9 shows the ICC (3, 1) for the criterion and estimation before and after training. All variables were calculated for the level surface trials, the sloped surface trials, and the overall trials. Figures 3 and 4 show the Bland-Altman plots between the criterion and estimated stroke number and push frequency from each accelerometer, respectively.

## 4. Discussion

This study provides insight into the usage of portable devices (e.g., triaxis accelerometers and wheel rotation monitor) to track upper extremity movements for wheelchair propulsion. The small discrepancies between the criterion and estimated parameters shown in Tables 4 and 5 suggest that wearable sensors have the potential to not only detect gross mobility levels of manual wheelchair users [20, 22, 35] but also to quantify the quality of upper extremity movements for wheelchair propulsion in terms of the repetitiveness.

In terms of estimating the stroke number and push frequency, the arm accelerometer showed the highest accuracy among the three accelerometers with a MAPE of 8.0% for stroke number and 12.9% for push frequency, indicating that the upper arm could be a good location for detecting temporal parameters of wheelchair propulsion. The wrist accelerometer showed higher MAPE than the arm accelerometer and this could be because the wrist accelerometer can be more sensitive to small upper extremity movements, possibly leading to the increased error. The seat accelerometer showed the lowest accuracy with a MAPE of 13.4% for the stroke number and 24.2% for the push frequency. The estimation errors for the seat accelerometer were greater than the study by Turner where an accelerometer was placed beneath the wheelchair seat to estimate the stroke number and push frequency among 10 manual wheelchair users. Unfortunately, the data analysis results were not described in detail. The study only reported an average percent error (i.e., −1.0% for stroke number and −1.7% for push frequency) instead of the MAPE averaged by each trial of each subject. An average percent error only indicates the estimation bias and may not be sufficient to show the estimation accuracy, as the positive and negative estimation errors from the trials may cancel each other, resulting in smaller overall errors [24].

Compared with the stroke number estimation, push frequency estimation was less accurate, which could be due to the estimation of the total cycle time comprised of push and recovery phases. The estimation algorithm based on the accelerometer signals was able to identify the push phase more accurately but unable to accurately determine the end of recovery phases, possibly leading to the inaccuracy when estimating the cycle time.

Tables 7 and 8 showed that subjects reduced the stroke number and push frequency after the propulsion training program, but there was only statistically significant difference on the push frequency along the upsloped surfaces. The accelerometers on the arm and wrist were also able to detect the difference. The ICC (3, 1) values in Table 9 also show that the accelerometers especially the ones on the arm and wrist were consistent with the criterion measures for detecting changes in stroke number and push frequency after the propulsion training. The responsiveness of the accelerometer and its estimation algorithm for propulsion parameters could potentially enable the evaluation of training interventions out of clinical settings, contributing to the preservation of upper limb functions of manual wheelchair users with SCI [36].

Considering the negative impact that upper extremity pain and injuries can have on manual wheelchair users with SCI, it is important to monitor and understand how the use of upper limbs during wheelchair propulsion and other ADLs are related to such pain and injury. The Clinical Practice Guideline on the Preservation of Upper Limb Function Following Spinal Cord Injury stresses the importance of reducing the frequency of repetitive upper limb tasks [15]. This study could result in a potential tool that can monitor the actual usage of upper extremities in terms of the repetitiveness during wheelchair propulsion and provide clinical professionals and researchers with an indication of activity levels as well as propulsion skills of manual wheelchair users in their daily life. Results in this study suggest that the use of accelerometers and wheel rotation monitors could potentially provide an objective measure of the repetitiveness of upper extremity movements of wheelchair users. This information may help clinicians to better understand and prevent upper extremity pain an injury among manual wheelchair users.

With the accelerometry technology getting cheaper and smaller, it is also possible to provide near real-time feedback to manual wheelchair users about their upper limb use and repetitiveness, further contributing to the prevention of upper limb pain and injury among this population. We envision the tools described in this study will be used during everyday living as follows: the wheelchair rotation monitor attached to the wheelchair wheel continuously monitors the wheelchair movement and determines the wheelchair propulsion episodes based on the wheelchair speed. If the wheelchair is determined to be moving continuously for a certain amount of time (e.g., 30 seconds), the accelerometer data for that period will be analyzed using the method described earlier, yielding the estimated stroke number and push frequency. The method can also accommodate the variations in propulsion style and speed within and between individual users by using their own movement data as reference. The estimated parameters could be used to provide feedback to the user in near real-time (if paired with a display or smartphone) or summary format to inform their progress over time. The push efficiency calculated by stroke number per feet or meter could also be obtained as an indicator of the user propulsion performance. The summary information could also help clinicians justify wheelchair prescription by knowing whether a user propels more efficiently using a specific type of wheelchair over another for a period of time at his/her home and community and evaluate the effect of interventions such as a propulsion training program or a new wheelchair/seating component. The devices could also support research that investigates the relationship between upper extremity usage and upper extremity pain and injury in a more accurate manner, contributing to our understanding of the etiology and prevention of upper extremity pain and injury prevalent in this population.

One limitation of the study is that the testing protocol was highly structured and involved only straight courses. Also using the wheel rotation monitor to identify self-propulsion episodes may not be accurate in real-life settings. Our previous study showed that the monitors we used here were able to detect self-propulsion, external pushing, sedentary activities, and other activities with an accuracy of 90% using a laboratory-based protocol [37]. Future testing should consider real-life testing with a mixture of wheelchair propulsion and other activities of daily living in the home and community settings and combine the detection of wheelchair episodes with the estimation of propulsion parameters when assessing the overall estimation accuracies of temporal propulsion parameters.

## 5. Conclusion

Results in this study suggest that the use of triaxis accelerometers and a wheel rotation monitor could be a viable option to accurately monitor temporal parameters of wheelchair propulsion such as stroke number and push frequency especially when the accelerometer is worn on the upper arm. This study could result in a potential tool that can monitor the actual usage of upper limbs in terms of the repetitiveness and contribute to the preservation of upper limb functions among manual wheelchair users with SCI.

## Figures and Tables

**Table 1:** Participant demographics.

Demographic variables	Mean ± standard deviation
Sex	
Female	6
Male	20
Age (years)	40 ± 14
Weight (lb.)	159 ± 41
Manual wheelchair usage (years)	13 ± 8
Injury level range	
Paraplegia (T4 and below)	20
Tetraplegia (T3 and above)	6
Self-reported pain (WUSPI)	7 ± 10

**Table 2:** Criterion and estimated stroke number.

	Video	Arm	Wrist	Seat
Level surface	24.6 ± 4.1	24.6 ± 4.0	24.6 ± 4.6	25.0 ± 4.3
Sloped surface	18.1 ± 1.1	17.2 ± 1.3	17.0 ± 1.4	17.7 ± 2.0
Overall	22.4 ± 3.6	22.2 ± 3.6	22.1 ± 4.0	22.6 ± 3.8

**Table 3:** Criterion and estimated push frequency (stroke/sec).

	SMART^Wheel^	Arm	Wrist	Seat
Level surface	0.95 ± 0.15	0.93 ± 0.09	0.94 ± 0.09	0.82 ± 0.19
Sloped surface	1.06 ± 0.09	1.02 ± 0.04	1.03 ± 0.13	0.94 ± 0.22
Overall	0.98 ± 0.11	0.96 ± 0.06	0.98 ± 0.09	0.86 ± 0.18

**Table 4:** Mean absolute error (MAE) and mean absolute percentage of error (MAPE) for the stroke number.

	MAE			MAPE %		
	ARM	WRIST	SEAT	ARM	WRIST	SEAT
Level surface	1.7 ± 1.5	2.4 ± 2.3	2.9 ± 3.5	7.7 ± 6.6	11.0 ± 10.2	13.5 ± 16.4
Sloped surface	1.5 ± 1.2	1.8 ± 1.3	2.4 ± 2.1	8.6 ± 7.0	10.3 ± 7.9	13.4 ± 11.8
Overall	1.6 ± 1.4	2.2 ± 2.1	2.7 ± 3.2	8.0 ± 7.1	10.8 ± 9.8	13.4 ± 15.6

**Table 5:** Mean absolute error (MAE) and mean absolute percentage of error (MAPE) for the push frequency.

	MAE			MAPE %		
	ARM	WRIST	SEAT	ARM	WRIST	SEAT
Level surface	0.1 ± 0.1	0.2 ± 0.2	0.3 ± 0.2	16.1 ± 16.7	21.5 ± 21.4	25.4 ± 16.9
Sloped surface	0.1 ± 0.1	0.1 ± 0.1	0.2 ± 0.2	6.4 ± 4.6	8.0 ± 6.1	21.8 ± 14.6
Overall	0.1 ± 0.1	0.1 ± 0.1	0.2 ± 0.2	12.9 ± 15.1	17.2 ± 19.3	24.2 ± 16.6

**Table 6:** Stroke number and push frequency intraclass correlation coefficient (ICC 3, 1).

		ICC	95% CI	*P* value
Stroke number	ARM	0.994	0.988~0.997	<0.001
	WRIST	0.990	0.980~0.995	<0.001
	SEAT	0.984	0.972~0.991	<0.001

Push frequency	ARM	0.916	0.843~0.953	<0.001
	WRIST	0.889	0.802~0.936	<0.001
	SEAT	0.690	0.071~0.868	<0.001

CI: confidence interval.

**Table 7:** Criterion and estimated stroke number before and after training, change, and *P* value.

		Stroke number				Change		*P* value
		Before		After				
		Mean		Mean		Mean		
Video	LS	25.5	±7.6	22.3	±5.7	−3.2	±0.96	0.09
	SS	18.2	±6.2	16.7	±4.4	−2.1	±1.13	0.68
	OA	21.8	±7.8	19.6	±5.8	−2.6	±1.02	0.16

Arm	LS	25.2	±7.2	22.7	±5.6	−2.5	±0.94	0.17
	SS	17.1	±5.9	15.8	±4.8	−1.9	±1.06	0.70
	OA	21.2	±7.7	19.3	±6.2	−2.2	±0.98	0.26

Wrist	LS	25.0	±7.0	23.2	±5.7	−1.8	±0.93	0.30
	SS	16.9	±6.0	16.1	±4.1	−1.4	±1.08	0.93
	OA	20.9	±7.7	19.7	±6.1	−1.6	±0.98	0.47

Seat	LS	26.3	±8.9	25.2	±6.4	−1.1	±0.84	0.12
	SS	18.0	±6.7	17.3	±5.6	−0.7	±1.06	0.66
	OA	22.1	±8.9	21.3	±6.8	−0.8	±0.95	0.19

LS: level surface propulsion trials, SS: sloped surface propulsion trials, and OA: level surface and sloped surface combined.

**Table 8:** Criterion and estimated push frequency before and after training, change, and *P* value.

		Push frequency (stroke/sec)				Change		*P* value
		Before		After				
		Mean		Mean		Mean		
SMW	LS	0.96	±0.16	0.88	±0.16	0.09	±0.12	0.06
	SS	1.13	±0.18	0.98	±0.15	0.18	±0.20	0.001
	OA	1.04	±0.19	0.93	±0.16	0.13	±0.17	0.001

Arm	LS	0.94	±0.14	0.89	±0.13	0.05	±0.13	0.197
	SS	1.05	±0.14	0.95	±0.13	0.14	±0.19	0.007
	OA	1.00	±0.15	0.92	±0.14	0.09	±0.17	0.007

Wrist	LS	0.93	±0.11	0.90	±0.12	0.03	±0.09	0.327
	SS	1.08	±0.19	0.98	±0.14	0.13	±0.21	0.024
	OA	1.00	±0.17	0.94	±0.13	0.08	±0.17	0.022

Seat	LS	0.84	±0.17	0.77	±0.14	0.07	±0.14	0.134
	SS	0.98	±0.30	0.87	±0.29	0.04	±0.40	0.081
	OA	0.91	±0.25	0.82	±0.19	0.06	±0.30	0.028

SMW: Smart^Wheel^, LS: level surface propulsion trials, SS: sloped surface propulsion trials, and OA: level surface and sloped surface combined.

**Table 9:** Stroke number and push frequency ICC (3, 1) before and after training.

		ICC	95% CI	*P* value
Stroke number	ARM	0.980	0.964~0.989	<0.001
	WRIST	0.969	0.916~0.986	<0.001
	SEAT	0.870	0.773~0.925	<0.001

Push frequency	ARM	0.856	0.684~0.899	<0.001
	WRIST	0.822	0.711~0.923	<0.001
	SEAT	0.568	0.248~0.752	<0.001

CI: confidence interval.
